# Neutralization of interleukin-9 ameliorates symptoms of allergic rhinitis by reducing Th2, Th9, and Th17 responses and increasing the Treg response in a murine model

**DOI:** 10.18632/oncotarget.15177

**Published:** 2017-02-07

**Authors:** Zhao Wei Gu, Yun Xiu Wang, Zhi Wei Cao

**Affiliations:** ^1^ Department of Otorhinolaryngology, China Medical University affiliated Shengjing Hospital, Shenyang, Liaoning, China; ^2^ Hematological Laboratory, China Medical University Affiliated Shengjing Hospital, Shenyang, Liaoning, China

**Keywords:** interleukin-9, neutralization, Th9, allergic rhinitis, murine model, Immunology and Microbiology Section, Immune

## Abstract

A novel independent Th-cell subset, characterized by high expression of interleukin (IL)-9, has been recognized as the Th9 subset. Although Th9 cells are important in many diseases, their contribution to allergic rhinitis (AR) remains unclear. We therefore first determined whether Th9 cells were present in a mouse model of AR. We then investigated the their involvement in the distribution of CD4^+^ T-cell subsets and the symptoms of AR by treating mice with anti-IL-9 antibodies (Abs). Anti-IL-9 Abs were administered intranasally during rechallenge of ovalbumin (OVA)-induced AR in BALB/c mice. We measured nasal rubbing motion, sneezing and eosinophils, as well as the Th1 (Th1 cell percentage, *Ifn-?* mRNA/protein, *T-bet* mRNA), Th2 (Th2 cell percentage, *Il-4* mRNA/protein, *Gata3* mRNA), Th9 (Th9 cell percentages *Il-9* mRNA/protein, *PU.1* and *Irf4* mRNA), Th17 (Th17 cell percentage, *Il-17* mRNA/protein, *Ror?t* mRNA), and Treg (Treg cell percentage, *Foxp3* mRNA) responses in the nasal mucosa. Treatment with anti-IL-9 Abs markedly reduced nasal rubbing, sneezing, eosinophil infiltration, and Th2, Th9, and Th17 responses, and increased the Treg response. Our findings emphasize the importance of IL-9/Th9 in the pathogenesis of AR, and suggest that anti-IL-9 Ab treatment may be an effective therapeutic strategy for AR.

## INTRODUCTION

Allergic rhinitis (AR) is one of the most prevalent airway diseases worldwide. Although AR is not life-threatening, it seriously reduces patients’ quality of life and work efficiency. AR may impair academic, work-related, emotional, and social function, so the great social and economic burden caused by AR should not be ignored [1–3].

AR is characterized by the involvement of CD4^+^ T-cell subsets and their associated immune mediators, including specific cytokines and chemokines [4, 5]. AR has long been thought to result from the upregulation of Th2 cells and a relative lack of Th1 cells [6]. Although the Th2 cell-mediated immune response explains many characteristics of AR, this simple model of inflammation is not sufficient to explain the immune mechanism behind AR. It was found that Th1 inflammation does not become dominant even when Th2 allergic inflammation is reduced [7], indicating that the pathogenesis of allergic disease is not entirely due to a Th1/Th2 imbalance, but is likely to involve other mechanisms, as well.

In recent years, the discovery of Th17 and regulatory T (Treg) cells introduced complexity into the existing Th1/Th2 balance paradigm and expanded our understanding of the pathogenesis of AR [4]. More recently, a novel, independent Th-cell subset (the ‘Th9’ subset), characterized by high expression of interleukin (IL)-9, was recognized [8–10]. IL-9 affects both inflammatory and normal tissue cells, increasing the numbers of lymphocytes, eosinophils and mast cells, stimulating IgE secretion, enhancing the responses of mast cells to allergens, promoting mucin expression, and stimulating cytokine secretion by inflammatory cells [11–14].

The various T-cell subsets regulate inflammatory responses mainly by secreting specific cytokines, such as interferon-γ (IFN-γ), IL-4, IL-5, IL-9, IL-13, and IL-17. Th9 cells were reported to be involved in the development of allergic asthma [15], although their precise contribution to AR remains unknown. Given that IL-9 has important functions in other inflammatory diseases, and that the use of anti-IL-9 antibodies (Abs) confers protective effects under these conditions [16–18], we reasoned that IL-9 or Th9 cells may be important contributors to AR, and that IL-9 neutralizing Ab therapy may therefore be effective against AR.

Thus, we first sought to evaluate the presence of Th9 cells and the distribution of CD4^+^ T-cell subsets in a mouse model of AR, and then investigated the involvement of IL-9 in the distribution of CD4^+^ T-cell subsets and the effects of IL-9 on AR by administering anti-IL-9 Abs.

## RESULTS

### Effect of IL-9 blockade on nasal symptoms in the AR mouse model

**Figure 1:**
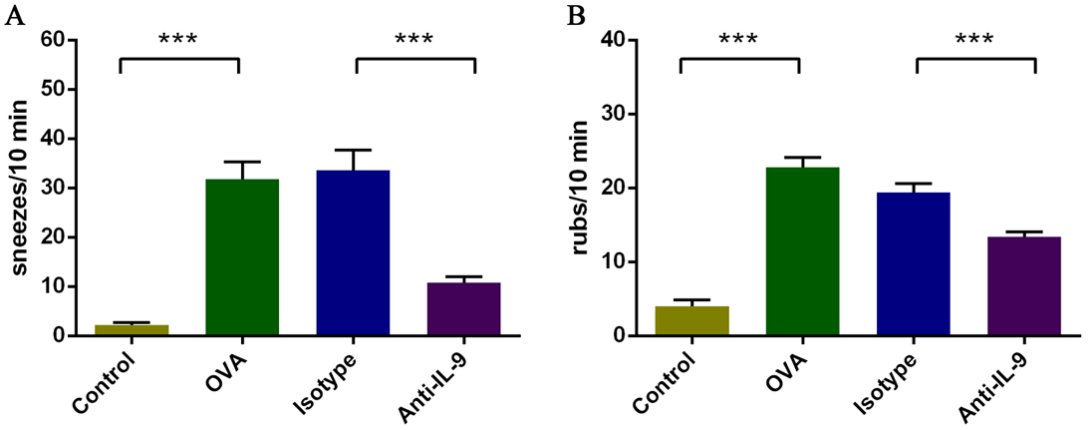
Inhibition of nasal symptoms resulting from IL-9 blockade **A**. Sneezing symptom score. **B**. Rubbing symptom score. ***P < 0.05.

To evaluate the effects of anti-IL-9 Ab treatment on AR symptoms in mice, we counted the numbers of sneezes and nasal rubs after the last OVA challenge (Figure 1). Mice in group B sneezed significantly more (31.8±7.79 sneezes/10 min) than those in group A (2.2±1.09 sneezes/10 min, *p* < 0.05). Mice treated with anti-IL-9 (group D) sneezed less (10.8±2.58 sneezes/10 min) than those in group C (33.6±9.21 sneezes/10 min, *p* < 0.05).

The effects on nasal rubbing were similar: 4.0±2.0 rubs/10 min for group A, 22.8±2.95 rubs/10 min for group B, 19.4±2.70 rubs/10 min for group C, and 13.4±1.52 rubs/10 min for group D. These results indicate that anti-IL-9 treatment can alleviate the symptoms of AR.

### Effect of IL-9 blockade on eosinophils

**Figure 2:**
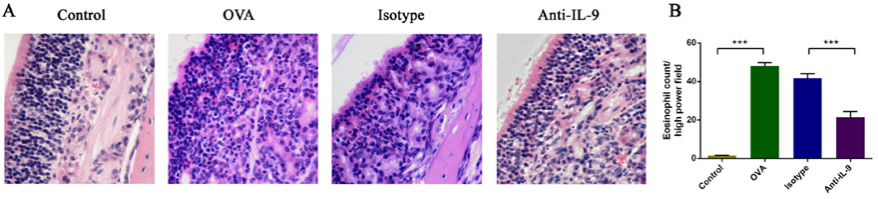
Anti-IL-9 Abs reduced the infiltration of eosinophils in the nasal mucosa Representative photomicrographs (original magnification ×200) of nasal mucosal sections from mice, stained with hematoxylin and eosin (H&E) for eosinophils **A**. Significantly greater numbers of eosinophils **B**. were observed in the OVA group than in the control group, and this increase was obviously alleviated by anti-IL-9 Ab treatment. ***P < 0.05.

Eosinophilic cytoplasm stained red with H&E (Figure 2A). H&E staining of the nasal mucosa revealed significantly greater infiltration of eosinophils in group B (47.95±4.21 cells/high-power field) than in group A (1.55±0.21 cells/high-power field; *p* < 0.05). In the mice treated with anti-IL-9 Abs, eosinophilic infiltration was significantly lower than in isotype-treated mice (21.55±6.46 cells/high-power field in group D *versus* 41.85±5.03 cells/high-power field in group C, *p* < 0.05) (Figure 2B).

These results indicate that anti-IL-9 Ab treatment can inhibit the infiltration of eosinophils.

### Effect of IL-9 blockade on cytokine levels in the nasal mucosa

**Figure 3:**
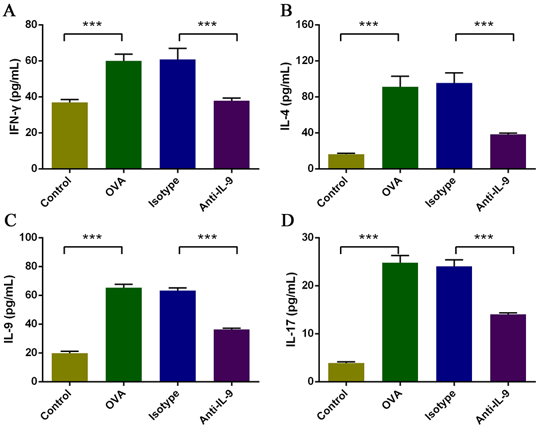
Anti-IL-9 reduced Th cell-related cytokine levels in the nasal mucosa The protein levels of IFN-γ, IL-4, IL-9, and IL-17 **A**., **B**., **C**., and **D**. were analyzed by CBA. The levels of all cytokines in the nasal mucosa were significantly greater in the OVA group than in the control group, and were markedly lower in the anti-IL-9-treated group than in the isotype-treated group. ***P < 0.05.

To evaluate the effects of anti-IL-9 Ab treatment on Th cell-related cytokine levels in the nasal mucosa, we analyzed the protein levels of IFN-γ, IL-4, IL-9, and IL-17 by CBA (Figure 3).

The levels of IFN-γ, IL-4, IL-9, and IL-17 in the nasal mucosa were significantly greater in the AR group (group B) than in the control group (group A; *p* < 0.05). The levels of these four cytokines were significantly lower in mice treated with anti-IL-9 Abs than in isotype-treated controls (group D *versus* group C; *p* < 0.05). IFN-γ levels were 36.9±3.8 pg/mL in group A, 60.0±8.3 pg/mL in group B, 60.7±13.9 pg/mL in group C, and 37.9±3.3 pg/mL in group D. IL-4 levels were 16.2±2.6 pg/mL in group A, 91.3±26.3 pg/mL in group B, 95.6±24.8 pg/mL in group C, and 38.5±2.9 pg/mL in group D. IL-9 levels were 19.8±3.0 pg/mL in group A, 65.3±5.2 pg/mL in group B, 63.4±3.8 pg/mL in group C, and 36.3±2.0 pg/mL in group D. IL-17 levels were 3.9±0.5 pg/mL in group A, 24.8±3.4 pg/mL in group B, 24.1±3.0 pg/mL in group C, and 14.0±0.7 pg/mL in group D.

Based on these results, the IL-9 blockade significantly attenuated the levels of IFN-γ, IL-4, IL-9, and IL-17. These data indicate that anti-IL-9 Abs suppress inflammation of the nasal mucosa by reducing the levels of multiple inflammatory mediators therein.

### Effect of IL-9 blockade on mRNA expression of Th cell-related cytokines and T-cell subset transcription factors in the nasal mucosa

**Figure 4:**
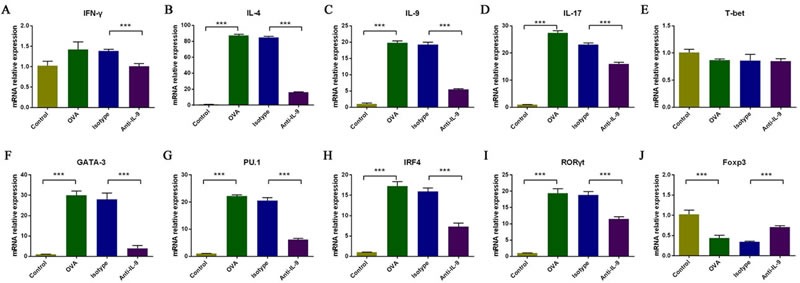
The effect of IL-9 blockade on the mRNA expression of Th cell-related cytokines and T-cell subset transcription factors in the nasal mucosa, analyzed with real-time PCR The relative levels of *Il-4*, *Il-9*, *Il-17*, *Gata3*, *PU.1*, *Irf4* and *Rorγt* mRNA **B**., **C**., **D**., **F**., **G**., **H**., and **I**. were significantly greater in the OVA group than in the control group, and this upregulation was markedly inhibited by anti-IL-9 Abs. *Ifn-γ* mRNA levels were moderately but not significantly greater in the OVA group than in the control group, but were significantly downregulated with anti-IL-9 Abs **A**. There was no significant difference in *T-bet* mRNA levels among the groups (E). *Foxp3* mRNA levels were significantly lower in the OVA group than in the control group, and were significantly upregulated with anti-IL-9 Abs **J**. ***P < 0.05.

To further evaluate the effects of anti-IL-9 Ab treatment on allergic inflammation, we performed real-time PCR to examine the mRNA expression of Th cell-related cytokines and T-cell subset transcription factors in the nasal mucosa (Figure 4). The mRNA levels of *Il-4*, *Il-9*, and *Il-17* were greater in group B than in group A (*p* < 0.05). Treatment with anti-IL-9 Abs significantly reduced the mRNA levels of *Il-4*, *Il-9*, and *Il-17* (group D *versus* group C; *p* < 0.05). The relative expression of *Il-4* mRNA was 1.02±0.26 in group A, 87.45±3.06 in group B, 84.92±2.78 in group C, and 16.38±0.92 in group D. The relative expression of *Il-9* mRNA was 1.03±0.26 in group A, 20.10±1.18 in group B, 19.50±1.73 in group C, and 5.57±0.46 in group D. The relative expression of *Il-17* mRNA was 1.02±0.10 in group A, 27.41±1.77 in group B, 23.15±1.23 in group C, and 15.94±1.43 in group D. *Ifn-γ* mRNA levels were slightly greater in group B than in group A, but not significantly so (*p* > 0.05). The mRNA level of *Ifn-γ* was significantly lower in mice treated with anti-IL-9 Abs than in isotype-treated controls (group D *versus* group C; *p* < 0.05). The relative expression of *Ifn-γ* was 1.02±0.24 in group A, 1.43±0.40 in group B, 1.38±0.10 in group C, and 1.00±0.14 in group D.

The mRNA levels of *Gata3*, *PU.1*, *Irf4* and *Rorγt* also were significantly higher in group B than in group A (*p* < 0.05) and significantly lower in group D than in group C (*p* < 0.05). The relative expression of *Gata3* mRNA was 1.02±0.23 in group A, 30.06±4.63 in group B, 28.03±6.90 in group C, and 3.84±0.15 in group D. The relative expression of *PU.1* mRNA was 1.02±0.22 in group A, 22.20±1.04 in group B, 20.56±2.36 in group C, and 6.23±0.78 in group D. The relative expression of *Irf4* mRNA was 1.00±0.03 in group A, 17.23±1.11 in group B, 15.94±0.83 in group C, and 7.33±0.81 in group D. The relative expression of *Rorγt* mRNA was 1.00±0.10 in group A, 19.39±3.05 in group B, 18.80±2.47 in group C, and 11.47±1.49 in group D. *T-bet* mRNA levels did not differ significantly among the four groups. The relative expression of *T-bet* mRNA was 1.00±0.13 in group A, 0.87±0.05 in group B, 0.86±0.25 in group C, and 0.85±0.10 in group D.

*Foxp3* mRNA expression was significantly lower in group B than in group A (*p* < 0.05). The mRNA levels of *Foxp3* were significantly greater in mice treated with anti-IL-9 Abs than in isotype-treated controls (group D *versus* group C; *p* < 0.05). The relative expression of *Foxp3* mRNA was 1.02±0.24 in group A, 0.44±0.15 in group B, 0.34±0.04 in group C, and 0.71±0.08 in group D.

These results suggest that anti-IL-9 Abs block allergic inflammation by inhibiting the mRNA expression of cytokines and specific transcription factors in Th2, Th9, and Th17 cells, and by increasing the expression of specific transcription factors in Tregs. The mRNA levels of Th1 cell-specific transcription factors and related cytokines do not seem to be affected.

### Effect of IL-9 blockade on T-cell subsets in the nasal mucosa

**Figure 5:**
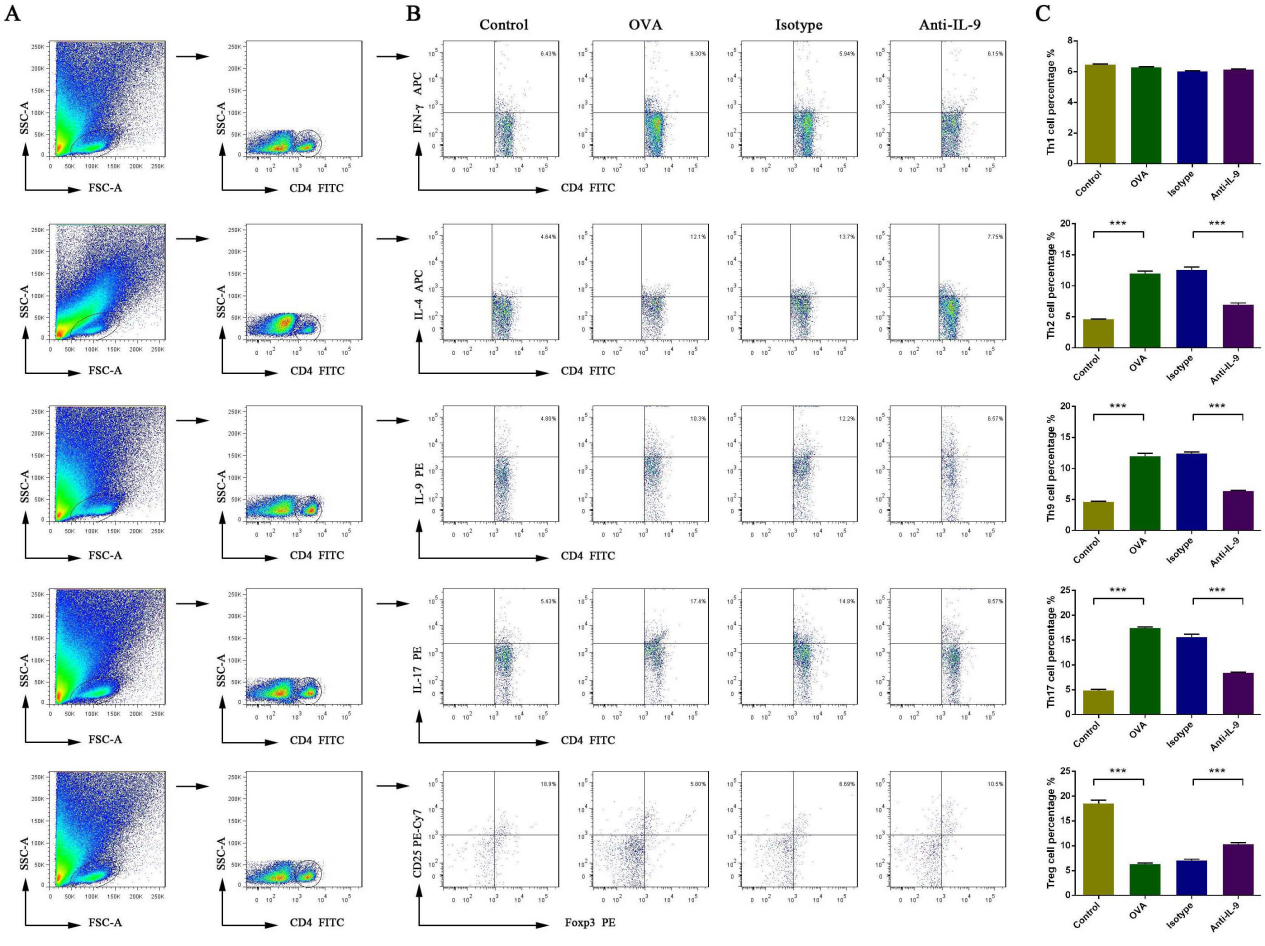
Effect of anti-IL-9 Ab treatment on the percentage of T-helper cell subsets and Treg cells in the nasal mucosa, analyzed by flow cytometry Gating of CD4^+^ T cell populations **A**.. Data are presented as representative results of four independent experiments **B**. and a statistical graph **C**. The numbers in the upper right quadrant are the percentages of T-helper cell subsets or Treg cells among CD4^+^ cells in gated populations of CD4^+^ T-cells (B). ***P < 0.05.

To evaluate the effects of anti-IL-9 Ab treatment on the lymphocytic immune response, we examined the percentages of T-helper cell subsets and Treg cells in the nasal mucosa using flow cytometry (Figure 5). The Th2, Th9, and Th17 cell percentages were greater in group B than in group A (*p* < 0.05). The percentages of these subsets were significantly lower in mice treated with anti-IL-9 Abs than in those treated with isotype Abs (group D *versus* group C; *p* < 0.05). The Th2 cell percentage was 4.58±0.15% in group A, 11.99±0.84% in group B, 12.55±1.00% in group C, and 6.97±0.61% in group D. The Th9 cell percentage was 4.60±0.27% in group A, 11.94±1.08% in group B, 12.44±0.48% in group C, and 6.34±0.32% in group D. The Th17 cell percentage was 4.87±0.43% in group A, 17.44±0.50% in group B, 15.60±1.37% in group C, and 8.37±0.35% in group D. The Th1 cell percentage did not differ significantly among the four groups. The Th1 cell percentage was 6.45±0.09% in group A, 6.27±0.10% in group B, 6.01±0.11% in group C, and 6.13±0.09% in group D.

The Treg cell percentage was lower in group B than in group A (*p* < 0.05). The Treg cell percentage was significantly greater in mice treated with anti-IL-9 Abs than in isotype-treated controls (group D *versus* group C; *p* < 0.05). The Treg cell percentage was 18.51±1.50% in group A, 6.31±0.55% in group B, 7.04±0.54% in group C, and 10.32±0.67% in group D.

## DISCUSSION

Recently, a novel, independent Th-cell subset (the ‘Th9’ subset) was recognized, which is characterized by high expression of IL-9. Th9 cells are involved in various forms of inflammation [15]. PU.1 and IRF4 are key proteins that contribute to the Th9 phenotype and are essential transcription factors in Th9 cells [10, 19]. Although Th9 cells are thought to function in many diseases, their effects in AR have not been clear. We demonstrated for the first time that the Th9 cell proportion was significantly greater in AR mice than in controls. The mRNA levels of *PU.1* and *Irf4* (encoding Th9 cell transcription factors) and the mRNA/protein levels of *Il-9* were also significantly elevated in AR. These results suggest that the Th9 response is central to the pathogenesis of AR.

AR is characterized by the accumulation of eosinophils and mast cells. Previous research has indicated that IL-9 affects both inflammatory and normal tissue cells, increasing the numbers of lymphocytes, eosinophils, and mast cells, enhancing the responses of mast cells to allergens, and promoting mucin expression [11–14]. Considering the effects of IL-9 on eosinophils and the important functions of eosinophils in AR, we blocked IL-9 to determine its influence on eosinophils in AR. The use of IL-9 neutralizing Abs significantly inhibited eosinophil infiltration, and symptoms improved significantly. Given their significant effects on AR symptoms, anti-IL-9 Abs have the potential to treat AR.

Th cells and their secreted cytokines are central promoters of the migration, apoptosis, and function of eosinophils and mast cells in AR [4]. Th1 cells secrete IFN-γ as part of the cellular immune response, while Th2 cells secrete IL-4, IL-5, and IL-13 as part of the humoral immune response. IL-5 can stimulate the development of eosinophils in the bone marrow and their exit into the circulation, while IL-4 and IL-13 can upregulate chemotactic factors like eotaxin to promote eosinophil infiltration.

Our knowledge of CD4^+^ T-cell differentiation has changed significantly, and new subsets, such as Th17 and Treg cells, continue to be recognized [17]. Importantly, the recent discovery of Th9 cells, the CD4^+^ T-cell subset that produces IL-9, has expanded significantly [20]. Under certain conditions, IL-9 can have pleiotropic effects on Th1, Th2, Th17, Treg, and Th9 cells, functioning in different ways depending on the microenvironment [21–27]. Considering the effects of IL-9 on Th cells and the important functions of Th cells in AR, we blocked IL-9 to determine its influence on the differentiation of Th-cell subsets in AR.

We found that the Th1 cell percentage and *T-bet* mRNA level did not differ significantly between AR mice and controls, and were unchanged by anti-IL-9 treatment during the OVA challenge. However, *Ifn-γ* protein and mRNA levels were greater in AR mice than in controls, and were downregulated by anti-IL-9 treatment during the OVA challenge. The apparent inconsistency between *Ifn-γ* and *T-bet* expression was not very surprising; it was reported that IFN-γ is secreted by various types of cells, including Th1 cells, macrophages, and epithelial cells, but T-bet is only expressed by Th1 cells [4]. This suggests that there is some other pathway that regulates IFN-γ, and that the Th1 response is not central to the pathogenesis of AR.

We found that Th2 responses (the Th2 cell percentage, *Il-4* mRNA/protein level, and *Gata3* mRNA level) were greater in AR mice than in controls and were downregulated by anti-IL-9 treatment, suggesting that anti-IL-9 Abs can suppress Th2 reactions. Indeed, inhibition of the Th2 response with anti-IL-9 Abs improved symptoms of AR.

The discovery of Th17 and Treg cells enhanced the understanding of the pathogenesis of AR. Th17 cells were first identified as IL-17-producing cells in 2000 [28], and RORγt, a transcription factor in mice, was discovered in 2006 [29]. Th17 cells have been associated with Th2-predominant allergic diseases. Increased Th17 responses have been found in asthma, AR, and nasal polyps [30–33]. Consistent with previous reports [4, 30], we found that Th17 responses (the Th17 cell percentage, *Il-17* mRNA/protein level, and *Rorγt* mRNA level) were greater in AR mice than in controls, and were downregulated with anti-IL-9 Ab treatment, suggesting that anti-IL-9 Abs can suppress Th17 reactions.

Treg cells express the specific transcription factor FOXP3 and are critical for preventing immune activation and suppressing inflammatory lesions [5]. Unlike other effector CD4^+^ T cells, Treg cells have been implicated in peripheral tolerance as inhibitors of immune responses. These cells suppress effector T cells of either the Th1 or Th2 phenotype when the latter types of cells are involved in inflammatory processes. Previous studies have found that *FOXP3* mRNA expression was reduced in AR subjects [34–36]. We found that Treg responses (Treg cell percentage, *Foxp3* mRNA) were lower in AR mice than in controls and were upregulated by anti-IL-9 Ab treatment. Anti-IL-9 treatment reportedly increases “signal transducer and activator of transcription” 5 (*STAT5)* expression and suppresses *STAT3* mRNA expression [26, 37]. STAT5 binds directly to the *FOXP3* gene and is required for optimal induction of FOXP3 *in vitro* [37,38], while STAT3 is required for IL-6-dependent downregulation of FOXP3 [38]. This suggests anti-IL-9 Abs increase the Treg cell number principally by regulating the STAT3 and STAT5 signaling pathways [37–40].

As noted, we found that Th9 responses (the Th9 cell percentage, *Il-9* mRNA/protein level, *PU.1* and *Irf4* mRNA levels) were greater in AR mice than in controls, while the increased Th9 responses were significantly downregulated by anti-IL-9 treatment in AR mice.

Our results indicate that the ratios of Th2, Th9, and Th17 cells increased, while the proportion of Treg cells decreased, in the AR state. After anti-IL-9 treatment, the proportion of Th2, Th9, and Th17 cells decreased, whereas the proportion of Treg cells increased, but the proportion of Th1 cells did not change significantly. Thus, in AR, Th2, Th9, Th17, and Treg cells are important components of lymphoid immunity.

It is accepted that Th9 cells are not the only source of IL-9; upon stimulation or under special circumstances, other cells have been reported to produce small amounts of IL-9, including natural killer T cells and type 2 innate lymphoid cells [41]. Our tentative identification of the source of IL-9 in the OVA-sensitized AR model is in agreement with the suggestions of Kaplan and Chang [42, 43]. However, the precise source of IL-9 should be confirmed in future work.

The downstream effects of IL-9 are primarily attributable to its receptor, IL-9R, which is expressed by effector but not naive T cells [44, 45]. Of the various Th-cell subsets, Th2 and Th17 cells maximally express IL-9R [46]. In patients with asthma, IL-9R is expressed by both lung mast and polymorphonuclear cells [47, 48]. However, the precise cellular targets of IL-9 in the OVA-induced AR mouse model remain unclear.

We found that IL-9 neutralization was associated with broad anti-inflammatory effects, and major changes in the Th1, Th2, Th17, or Treg response clearly alleviated allergic symptoms in the AR model. These data indicate that Th-cell subsets do not operate independently; rather, they appear to work co-operatively and network. IL-9 is a key component of this process, so inhibiting this protein may be useful for the treatment of AR.

Our findings are limited to the AR model induced by OVA. It is unclear what effect anti-IL-9 Abs would have on AR mouse models sensitized by other antigens, such as house dust mites. In addition, Treg cells mainly secrete IL-10 and TGF-β. It is worth noting that IL-4 can represent the Th2 subgroup, while IL-9 can represent the Th9 subgroup. However, IL-10 and TGF-β did not represent the Treg subgroup.

It is noteworthy that because IL-4 was produced primarily by the Th2 subgroup, it can reflect the function of Th2. Similarly, IL-9 was produced primarily by the Th9 subgroup, enabling it can reflect the function of Th9. However, Treg cells mainly secrete IL-10 and TGF-β. IL-10 and TGF-β were not produced primarily by the Treg subgroup. The present study therefore does not involve cytokines from Treg cells.

In conclusion, we have shown for the first time that Th9 cells are involved in AR development, and that neutralization of IL-9 has broad anti-inflammatory effects. We expect that our results will improve the understanding of the pathogenesis of AR and facilitate the development of novel antibody-based therapies for the management of this disease.

## MATERIALS AND METHODS

### Animals

Eight-week-old female BALB/c mice, free of murine-specific pathogens, were obtained from the animal department at Shengjing Hospital, China Medical University (Shenyang, China). The mice were housed in a controlled environment with a 12/12-h light/dark cycle with free access to food and water. They were maintained on an ovalbumin (OVA)-free diet. The experimental procedures were approved by the ethical committee of Shengjing Hospital, China Medical University.

### Sensitization and antigen challenge

BALB/c mice were divided into four treatment groups of 10 mice each as follows: (1) control group mice, sensitized and challenged with saline (group A), (2) OVA group mice, sensitized and challenged with OVA (group B), (3) isotype group mice, treated with isotype Abs for anti-IL-9 (group C), and (4) anti-IL-9 group mice, treated with an anti-IL-9 Ab (group D).

The OVA group mice were sensitized on days 0, 2, 4, 6, 8, 10, 12, and 14 by intraperitoneal (i.p.) injection with 1 mg/mL OVA (Sigma-Aldrich, St. Louis, MO, USA) and 20 mg/mL aluminum hydroxide (Sigma-Aldrich) in saline at a dose of 100 μL/mouse.

After 2 weeks, the animals were challenged by daily nasal instillation of 100 μg OVA in 20 μL saline per mouse, by means of a micropipette, from day 15 to day 25.

Mice from the isotype group and the anti-IL-9 group were given intranasal instillations of hamster IgG (isotype Ab for anti-IL-9, eBioscience, San Diego, CA, USA) and anti-IL-9 Abs (eBioscience), respectively, 30 min prior to the OVA challenge, at a dose of 10 μg in 20 μL saline per mouse. Control group mice were sensitized and challenged with saline instead of OVA at all stages.

### Evaluation of allergic symptoms induced after allergen challenge

On day 25, during the 10-min period after the final intranasal OVA administration, the numbers of nasal rubbing motions and sneezes were recorded by four observers who were blinded to the experimental groups. The average values of the four observers’ measurements were used for statistical analysis.

### Histopathology

Two hours after the last challenge on day 25, the mice were sacrificed. The heads of five mice per group were removed and immersed in 4% buffered paraformaldehyde. After fixation, the heads were decalcified in 10% ethylenediamine tetraacetic acid for 28 days. The specimens were embedded in paraffin wax and sectioned coronally at a thickness of 4 μm. The sections were stained with hematoxylin and eosin (H&E) for eosinophils.

In four randomly selected fields from each section, the numbers of eosinophils in the nasal mucosa were counted under a microscope at ×200 magnification, and the mean value for each field was calculated. The counts were made by two observers who were blinded to the treatments.

### Collection of nasal mucosa

The nasal mucosae of the remaining five mice in each group were obtained. The nasal mucosae were divided into two parts: one part was prepared into a single-cell suspension and centrifuged. After centrifugation, the supernatant was stored at -80°C for cytokine measurement, while the pellet was used for Th1, Th2, Th9, Th17, and Treg cell percentage analysis. The second part was immediately snap-frozen in liquid nitrogen and stored at -80°C for RNA extraction.

### RNA extraction/reverse transcription and real-time polymerase chain reaction (PCR)

Total RNA was extracted with the TRIzol reagent (Invitrogen, Shanghai, China), and total RNA (0.5 µg) was reverse-transcribed to complementary DNA with a PrimeScript RT kit (Takara, Dalian, China), according to the manufacturer's protocol. Real-time PCR was performed with SYBR Premix Ex Taq (Takara) on an ABI 7500 Real-time PCR System (Applied Biosystems, Foster City, CA, USA).

*β-actin* (a housekeeping gene) served as a control, while *Ifn-γ*, *Il-4*, *Il-9*, *Il-17a*, *T-bet*, *Gata3*, *PU.1*, interferon regulatory factor 4 (*Irf4*), *Rorγt*, and *Foxp3* were the target genes. The following primer sequences were used (all 5′ to 3′): forward primer GCAGAAGGAGATTACTGCTCT, reverse primer GCTGATCCACATCTGCTGGAA for *β-actin*; forward primer CTGCTGATGGGAGGAGATGT, reverse primer TTTGTCATTCGGGTGTAGTCA for *Ifn-γ*; forward primer TGTACCAGGAGCCATATCCA, reverse primer TGTTCTTCGTTGCTGTGAGG for *Il-4*; forward primer GGGCATCAGAGACACCAAT, reverse primer GGACGGAGAGACACAAGCA for *Il-9*; forward primer TCTCTGATGCTGTTGCTGCT, reverse primer CGTGGAACGGTTGAGGTAGT for *Il-17a*; forward primer TACAACAGCCAGCCAAACAG, reverse primer CACCCTTCAAACCCTTCCTC for *T-bet*; forward primer TACCACCTATCCGCCCTATG, reverse primer GCCTCGACTTACATCCGAAC for *Gata3*; forward primer CTTCCAGTTCTCGTCCAAGC, reverse primer TTCTTCACCTCGCCTGTCTT for *PU.1*; forward primer ACTTGCCTTCACAACCGTCT, reverse primer CCCGAAAGAGTCAGGAATGA for *Irf4*; forward primer AGCCTTTCCCTTTCTGCACT, reverse primer CCATCACTTGCTGCTGTTGT for *Rorγt*; and forward primer GCCAAGCAGAAAGATGACAG, reverse primer TTCCAGATGTTGTGGGTGAG for *Foxp3*.

mRNA levels were measured by the cycle threshold (2^-ΔΔCT^) method and were normalized to *β-actin* levels. A no-template sample served as a negative control.

### Measurement of cytokine concentrations

Levels of IFN-γ, IL-4, IL-9, and IL-17A in supernatants were measured with a Cytometric Bead Array (CBA) Flex Set (BD Biosciences, San Jose, CA, USA) according to the manufacturer's instructions. Briefly, four capture bead populations that had distinct fluorescence intensities and were coated with cytokine-specific capture Abs were mixed together in equal volumes; then, 50 μL of each sample and 50 μL of phycoerythrin (PE)-conjugated detection Abs were added to 50 μL of the mixed-bead population. Each mixture was incubated for 3 h at room temperature in the dark so that sandwich complexes could form. Then, the beads were washed with wash buffer, and data were acquired with a BD FACSCanto II flow cytometer (BD Biosciences). FACSDiva and BD CBA software 4.2 (BD Biosciences) were used for the analyses.

The limits of detection were 0.5 pg/mL for IFN-γ, 0.3 pg/mL for IL-4, 10.7 pg/mL for IL-9, and 0.95 pg/mL for IL-17A. Zero values were assigned when levels were under these limits.

### Flow cytometric analysis of Th1, Th2, Th9, and Th17 cell percentages

Cells were stimulated with 50 ng/mL phorbol myristate acetate (Sigma-Aldrich, St. Louis, MO, USA), 1 µg/mL ionomycin (Sigma-Aldrich), and 10 µg/mL GolgiStop (BD Biosciences) at 37°C under 5% (v/v) CO_2_ for 6 h, then stained with fluorescein isothiocyanate-labeled anti-CD4 Abs (BD Biosciences) and fixed and permeabilized with a fix/perm solution (eBioscience, San Diego, CA, USA) according to the manufacturer's instructions. The cells were then incubated with an allophycocyanin (APC)-labeled IFN-γ Ab, APC-labeled IL-4 Ab, PE-labeled IL-9 Ab, and PE-labeled IL-17A Ab (BD Biosciences). Cells in the control group were stained with the isotype control. Samples were analyzed on a BD FACSCanto II flow cytometer (BD Biosciences), and data were evaluated with FlowJo software (ver. 7.6; TreeStar Inc., San Carlos, CA, USA).

### Flow cytometric analysis of Treg cell percentages

Cells were stained with a fluorescein isothiocyanate-labeled CD4 Ab and a phycoerythrin-cyanin 7 (PE-Cy7)-labeled CD25 Ab (BD Biosciences) and were fixed and permeabilized with a fix/perm solution (eBioscience) according to the manufacturer's instructions. The cells were then incubated with a PE-labeled FOXP3 Ab (BD Biosciences), and those in the control group were stained with a FOXP3 isotype control. Samples were analyzed on a BD FACSCanto II flow cytometer (BD Biosciences), and data were evaluated with FlowJo software (ver. 7.6; TreeStar Inc.).

### Statistical analysis

All results are expressed as the mean ± SEM. Results from the different groups were compared through the non-parametric Kruskal-Wallis test, followed by the Mann-Whitney U-test. Statistical analyses were performed with SPSS software (ver. 13.0; SPSS Inc., Chicago, IL, USA). *P* values < 0.05 were considered to indicate statistical significance.
